# Incomplete recovery from rocuronium-induced muscle relaxation in patients with amyotrophic lateral sclerosis using sugammadex: A case report

**DOI:** 10.1097/MD.0000000000018867

**Published:** 2020-01-17

**Authors:** Hea Rim Chun, Jinhun Chung, Nan Seol Kim, A. Joo Kim, Suro Kim, Kyu Sik Kang

**Affiliations:** Department of anesthesiology and pain medicine, Soonchunhyang University Hospital Cheonan, 31, Soonchunhyang 6gil, Dongnam-gu, Cheonan, Chungcheongnam-do, Korea.

**Keywords:** amyotrophic lateral sclerosis, muscle relaxation, sugammadex

## Abstract

**Introduction:**

Patients with motor neuron diseases, such as amyotrophic lateral sclerosis (ALS), have higher sensitivity to nondepolarizing neuromuscular blocking agents (NMBAs) and are at higher risk for a residual block. For this reason, the use of NMBAs such as rocuronium has been limited owing to the delayed reversal of muscle relaxation. It was recently reported that rapid and effective reversal of muscle relaxation occurs when sugammadex, a muscle relaxant reversal drug, is administered to patients in ALS with rocuronium-induced muscle relaxation. However, in this paper, we report the incomplete recovery and recurarization of muscle relaxation after sugammadex administration in ALS patients, and delayed recovery of muscle relaxation after additional administration of sugammadex.

**Patient concerns:**

A 71-year-old male patient with ALS received general anesthesia for laparoscopic nephroureterectomy.

**Diagnosis:**

The patient was diagnosed with ALS 2 years earlier, and scheduled to undergo laparoscopic nephroureterectomy for ureteral cancer.

**Intervention:**

We used sugammadex for the reversal of deep neuromuscular block. We measured a train-of-four (TOF) count of 4 and a TOF ratio of 54% at about 8 min after administration of 4 mg/kg sugammadex. However, then the TOF count decreased to 1 to 3 and tidal volume (TV) decreased to < 100 mL. Therefore, an additional 50 mg sugammadex was administered intravenously 12 min after the first dose of sugammadex was injected.

**Outcomes:**

The patient's vital signs were stable and his recovery from anesthesia was uneventful. Therefore, he was discharged to the intensive care unit. The patient had aspiration pneumonia symptoms owing to dysphagia on the third postoperative day, but after the symptoms improved he was transferred to the hospital for rehabilitation of dysphagia and dyspnea.

**Conclusion:**

It is critical to monitor whether muscle relaxation is sufficiently reversed when using sugammadex in ALS patients. Further research is needed to determine the appropriate dose of sugammadex for muscle relaxation reversal.

## 1 Introduction

Amyotrophic lateral sclerosis (ALS) is the most common form of motor neuron disease caused by the progressive degeneration of motor neurons. Its clinical features include atrophy, weakness, and fasciculation of the affected limb. If the motor neurons of the bulbar muscles are involved, symptoms such as dysphagia, dysarthria, and dyspnea may be present.^[1]^

In the past, the use of neuromuscular blocking agents during laparoscopy-assisted abdominal surgery was contraindicated or required extra caution in patients with neuromuscular diseases such as myasthenia gravis (MG) and ALS. This is because the use of succinylcholine increases the risk for hyperkalemia and arrest,^[2]^ and the use of nondepolarizing neuromuscular blocking agents (NMBAs) increases the risk of residual block and subsequent respiratory failure owing to increased sensitivity to the drug,^[3]^ leading to an extremely challenging situation for the anesthesiologist.

However, the development of sugammadex (Bridion), which reverses muscle relaxation induced by aminosteroid NMBAs such as rocuronium or vecuronium, significantly reduces the incidence of residual block in 20% to 40% of normal individuals,^[4]^ and there have been several reports that sugammadex reverses muscle relaxation safely and effectively in MG patients.^[5–7]^

In addition, some studies have reported that sugammadex is effective for patients with ALS.^[8–10]^ However, we report a case of an ALS patient who received sugammadex after being given rocuronium for muscle relaxation during general surgery; the patient experienced delayed recovery of muscle relaxation and recurarization.

## 2 Case report

Written informed consent was obtained from the patient for the publication of this report. The patient, who was diagnosed with ALS 2 years earlier, was a 71-year-old man scheduled to undergo laparoscopic nephroureterectomy for ureteral cancer.

The patient complained of dysphagia, dysarthria, and dyspnea at the time of admission. The motor power grade of the both upper extremity were 3, and the both lower extremity were 4. The patient had a history of hypertension and was taking 100 mg riluzole (Uriteck) for the treatment of ALS. The patient's weight was 54 kg. The chest radiography showed normal findings; however, in pulmonary function tests, his functional vital capacity (FVC) was 42%, forced expiratory volume in 1 s (FEV1) was 47%, and FEV 1/FVC was 73%. This indicated pulmonary dysfunction with a severe restrictive pattern. Premedication consisted of 0.1 mg glycopyrrolate administered intramuscularly. Upon arrival in the operating room, standard intraoperative monitoring procedures including electrocardiogram, pulse oximetry, and noninvasive arterial blood pressure were performed.

Initial oxygen saturation (SpO_2_) was 97%. To confirm the degree of neuromuscular block (NMB), train-of-four (TOF) stimuli were applied to the ulnar nerve using a neuromuscular transmission module (M-NMT Module, Datex-Ohmeda Inc, Helsinki, Finland).

After preoxygenation with 100% oxygen, anesthesia was induced using 2 mg/kg (100 mg) 1% propofol and maintained with desflurane at 5 vol%. The initial TOF ratio was 94% and the TOF count was 4 before the patient received an IV bolus injection of 0.6 mg/kg (30 mg) rocuronium bromide (Rocnium). After endotracheal intubation, the lungs were ventilated with a mixture of oxygen and air at a ratio of 1:2 and left radial artery cannulation after a modified Allen test was performed to monitor invasive blood pressure. The operation commenced 30 min after induction; 70 min after induction, 5 mg rocuronium bromide was injected intravenously because the TOF count and ratio were 4% and 54%, respectively. The durations of the operation and anesthesia were 3 and 3 h 40 min, respectively. At the end of the procedure, the TOF count was 0 and the post-tetanic count (PTC) was 1, indicating that the patient was in a deep NMB state. Reversal of rocuronium-induced NMB was performed by administering 4.0 mg/kg (200 mg) sugammadex. After 3 min and 20 s we obtained a TOF count of 3, and after 7 min and 40 s we obtained a TOF count and ratio of 4% and 73%, respectively. However, then the TOF ratio decreased to 54% and tidal volume (TV) was less than 100 mL.

Then, the TOF count decreased to 1 to 3, and we decided to add 50 mg sugammadex 12 min after initial sugammadex administration. Three minutes later, TV was 250 mL. After a further 40 s, the TOF count and ratio were 4% and 144%, respectively, and regular respiration was observed. Therefore, tracheal extubation was performed.

The patient's vital signs were stable and his recovery from anesthesia was uneventful. Therefore, he was discharged to the intensive care unit. The patient had aspiration pneumonia symptoms owing to dysphagia on the third postoperative day, but after the symptoms improved he was transferred to the hospital for rehabilitation of dysphagia and dyspnea.

## 3 Discussion

ALS is the most common form of motor neuron disease caused by the progressive degeneration of motor neurons. It occurs in 4 to 8 per 100,000 individuals and can invade both lower and upper motor neurons. In 80% of patients, the first effects occur in a limb or the spinal cord, while in 20% of patients the disease affects the motor neurons of the brainstem. Patients with brainstem lesions exhibit symptoms such as dysphagia, dysarthria, and dyspnea in the early stages, and the disease progresses rapidly.^[1]^ The course of the disease varies according to the first affected region and the clinical manifestation, and usually respiratory failure is the cause of death.^[11]^

Abdominal surgery using laparoscopy is safe when undergoing general anesthesia with sufficient muscle relaxation. However, in patients with motor neuron diseases such as ALS, hyperkalemia and cardiac arrest have been reported when succinylcholine is used.^[2]^ The use of NMDAs such as rocuronium in patients with ALS is reported to delay the recovery of muscle relaxation and increase sensitivity to NMDAs,^[3]^ for example in MG patients.^[12]^

Sugammadex is a modified γ-cyclodextrin that encapsulates aminosteroid NMBAs such as rocuronium and vecuronium and reverses muscle relaxation by rapidly reducing the concentration of NMBAs in the plasma.^[13,14]^ Its effects are dose-dependent. In normal individuals, 2 mg/kg is recommended for moderate NMB and 4 mg/kg for deep blockade with a TOF count of 0, and it is recommended to use 4 to 8 mg/kg in cases of profound block.^[14]^ In patients with muscle diseases, such as myotonias and dystonias, it is recommended to administer sugammadex at the same dose as in normal individuals.^[13]^ Vymazal et al reported that administering 2 to 4 mg/kg sugammadex to 117 patients with MG effectively reversed muscle relaxation.^[5]^

However, case reports of the use of rocuronium and sugammadex in patients with ALS are rare. In two cases, rocuronium was administered for muscle relaxation in ALS patients. The TOF ratio was measured to be 0.9 or higher at the end of the operation, but the patients’ TVs were insufficient and the patients complained of dyspnea and muscle weakness. Thus, the authors used sugammadex 1 to 2 mg/kg for full recovery of muscle relaxation.^[8,9]^

Yoo et al used rocuronium for muscle relaxation and reported that the TOF count was 0 and the PTC was 2 at the end of the operation. Thus, they used 5 mg/kg sugammadex to reverse muscle relaxation and safely performed tracheal extubation at 4 min after administration.^[10]^

In the present report, unlike other cases where full recovery occurred within 3 to 5 min,^[8–10]^ we administered 4 mg/kg sugammadex when the TOF was 0; the TOF count was 4 after 8 min. After delayed recovery of muscle relaxation, recurarization occurred and full recovery was achieved after adding 1 mg/kg sugammadex.

Residual block causes not only discomfort owing to muscle weakness but also fatal respiratory complications such as airway obstruction and hypoxemia.^[15]^ Therefore, during general anesthesia with a muscle relaxant, tracheal extubation is recommended until muscle relaxation is completely recovered. A TOF ratio above 0.9 is an objective and reliable indicator of complete recovery of muscle relaxation.^[4]^

Inadequate recovery is associated with a TOF ratio of 0.9 or less and has been reported in 20% to 40% of patients in post-anesthetic care units when anticholinesterase, a conventional reversal agent of NMDA, is used.^[4]^ The incidence of residual blockade decreases to about 1.15% when sugammadex is administered,^[16]^ and it has also been reported that residual blockade does not occur when 2 mg/kg sugammadex is administered in moderate NMB, and 4 mg/kg in deep NMB, with a TOF count of 0 and PTC ≥ 1.^[17]^

Because sugammadex encapsulates aminosteroid NMBA at a 1:1 ratio, the concentration of aminosteroid NMBA is an important factor in the reversal of muscle relaxation.^[4]^ Residual NMB may occur after the administration of sugammadex when the depth of muscle relaxation is not monitored^[16]^ or when sugammadex is not administered at the appropriate dose for the degree of muscle relaxation.

In our case, a neuromuscular transmission module was used to measure the degree of muscle relaxation and 4 mg/kg sugammadex, the dose used to reverse the deep blockade in general, was used. However, a residual blockade appeared. In our patient, dysphagia was worse postoperatively, suggesting that muscle weakness caused by rocuronium was accompanied by weakness of the pharyngeal muscle. Muscle relaxation was completely reversed after adding sugammadex. Considering these points, as in the cases of Kelsaka et al^[8]^ and Chang et al,^[9]^ the dose of sugammadex appears to have been inadequate because the patient was in a deeper state of muscular blockade than the degree of muscle relaxation measured with the neuromuscular transmission module.

Although several studies have suggested that sugammadex is safe in patients with neuromuscular disease when used at normal doses, we recommend carefully determining the dose based on the progression of the individual's disease, and then monitoring the degree of recovery of muscle relaxation. Further research is needed to determine the optimal dose depending on these and other parameters.

## Author contributions

**Conceptualization:** Hea Rim Chun, Jinhun Chung.

**Investigation:** A Joo Kim, Suro Kim.

**Supervision:** Jinhun Chung, Nan Seol Kim, Kyu Sik Kang.

**Writing – original draft:** Hea Rim Chun, A Joo Kim.

**Writing – review & editing:** Hea Rim Chun, Nan Seol Kim, Kyu Sik Kang.

Hea Rim Chun orcid: 0000-0003-3655-9052.
